# Impairment of alternative splice sites defining a novel gammaretroviral exon within *gag *modifies the oncogenic properties of Akv murine leukemia virus

**DOI:** 10.1186/1742-4690-4-46

**Published:** 2007-07-06

**Authors:** Annette Balle Sørensen, Anders H Lund, Sandra Kunder, Leticia Quintanilla-Martinez, Jörg Schmidt, Bruce Wang, Matthias Wabl, Finn Skou Pedersen

**Affiliations:** 1 Department of Molecular Biology, University of Aarhus, Denmark; 2 Institute of Pathology, GSF-National Research Center for Environment and Health, Neuherberg, Germany; 3 Department of Comparative Medicine GSF-National Research Center for Environment and Health, Neuherberg, Germany; 4 Picobella, Burlingame, CA, USA; 5 Department of Microbiology and Immunology, University of California-San Francisco, San Francisco, CA, USA; 6 The State and University Library, Universitetsparken, DK-8000 Aarhus C, Denmark; 7 Biotech Research and Innovation Centre (BRIC), University of Copenhagen, Ole Maaløes Vej 5, DK-2200 Copenhagen N, Denmark

## Abstract

**Background:**

Mutations of an alternative splice donor site located within the *gag *region has previously been shown to broaden the pathogenic potential of the T-lymphomagenic gammaretrovirus Moloney murine leukemia virus, while the equivalent mutations in the erythroleukemia inducing Friend murine leukemia virus seem to have no influence on the disease-inducing potential of this virus. In the present study we investigate the splice pattern as well as the possible effects of mutating the alternative splice sites on the oncogenic properties of the B-lymphomagenic Akv murine leukemia virus.

**Results:**

By exon-trapping procedures we have identified a novel gammaretroviral exon, resulting from usage of alternative splice acceptor (SA') and splice donor (SD') sites located in the capsid region of *gag *of the B-cell lymphomagenic Akv murine leukemia virus. To analyze possible effects *in vivo *of this novel exon, three different alternative splice site mutant viruses, mutated in either the SA', in the SD', or in both sites, respectively, were constructed and injected into newborn inbred NMRI mice. Most of the infected mice (about 90%) developed hematopoietic neoplasms within 250 days, and histological examination of the tumors showed that the introduced synonymous *gag *mutations have a significant influence on the phenotype of the induced tumors, changing the distribution of the different types as well as generating tumors of additional specificities such as *de novo *diffuse large B cell lymphoma (DLBCL) and histiocytic sarcoma. Interestingly, a broader spectrum of diagnoses was made from the two single splice-site mutants than from as well the wild-type as the double splice-site mutant. Both single- and double-spliced transcripts are produced *in vivo *using the SA' and/or the SD' sites, but the mechanisms underlying the observed effects on oncogenesis remain to be clarified. Likewise, analyses of provirus integration sites in tumor tissues, which identified 111 novel RISs (retroviral integration sites) and 35 novel CISs (common integration sites), did not clearly point to specific target genes or pathways to be associated with specific tumor diagnoses or individual viral mutants.

**Conclusion:**

We present here the first example of a doubly spliced transcript within the group of gammaretroviruses, and we show that mutation of the alternative splice sites that define this novel RNA product change the oncogenic potential of Akv murine leukemia virus.

## Background

Many murine leukemia viruses (MLVs) belonging to the genus gammaretroviruses induce cancer when injected into susceptible newborn mice [1,2]. These simple retroviruses do not themselves harbor transduced oncogenes, and their ability to cause cancer relies on the host cellular genes that are transcriptionally activated or otherwise mutated as a result of the integrated provirus [3-6].

Regarding the virus itself, it is well documented that the LTR region plays a crucial role for both the strength and cell type specificity of disease induction [7,8]. Within the LTR the specificity has been located mainly to the enhancer region in U3, and further narrowed down to the sequences defining different transcription factor binding sites [9-12]. In spite of this predominant role of the LTR in MLV pathogenesis, also sequences outside this region have been shown to be important for the ability and potency of a particular virus to induce cancer. Infection is mediated by interaction between the viral envelope protein (Env) and a specific host cell receptor, and for the ecotropic MLVs such as Moloney, Akv, and SL3-3, this receptor has been identified as the mouse cationic amino acid transporter 1 (mCAT1) [13,14]. A significant role of *env *in MLV pathogenesis is the involvement in the generation of recombinant polytropic viruses that takes place during T-cell lymphoma development. These MCF (mink cell focus-forming) viruses have the ability to superinfect cells, an aspect which is thought to contribute to tumor formation [15,16]. In addition to the *env *gene, and perhaps somewhat surprisingly, the viral *gag *gene sequences have also proven to play a role in MLV pathogenesis. Thus, Audit *et al*. (1999) [17] showed that the introduction of only three synonymous nucleotide mutations in the capsid-coding gene of Moloney MLV (Mo-MLV) changed the oncogenic properties of this virus. The mutations were located at an alternative splice donor site (SD'), which together with the canonical *env *splice acceptor site was shown to produce a subgenomic transcript of 4.4 kb [18]. The equivalent transcript, produced by Friend MLV, was subsequently shown to be packaged into virions, reversely transcribed and integrated in the host genome by normal viral mechanisms [19]. While wild-type Mo-MLV induces T-cell lymphomas in 100% of the inoculated mice, the SD' mutant virus exhibited a much broader specificity, thus inducing – besides the expected T-cell tumors – erythroid or myelomonocytic leukemias. In contrast, the corresponding mutations in a Friend MLV background did not seem to influence the pathogenic potential of this virus at all. Both wild-type and mutant Friend MLVs induced exclusively the characteristic erythroleukemia [17]. So it seems that the importance for the disease-inducing potential of the SD' site, although conserved among many species, is strongly dependent on the virus type.

The SD' site has also been found to be used for production of the oncogenic *gag-myb *fusion RNAs in promonocytic leukemias induced by Mo-MLV in pristane-treated BALB/c mice [20]. When the SD' site was mutated in this model, the overall disease incidence was not affected; however the proportion of myeloid leukemia decreased significantly, while the proportion of lymphoid leukemia increased. Moreover, no 5' insertional activation of c-*myb *(using alternative splice donor sites) could be found, thereby signifying a specific requirement of the SD' site for this mechanism [21].

Here we report of the identification of an alternative splice acceptor site, SA', located in the capsid region of *gag*, which together with the *gag *splice donor site, SD' (corresponding to the one reported for Moloney and Friend MLV), or together with a second alternative *gag *splice donor site, SD*, defines a novel exon within the genus gammaretroviruses. We show that RNA splicing by use of the alternative splice sites does indeed take place in tumor tissue, and that both double- and single-spliced transcripts are produced. When mutating the SD', the SA', or both sites simultaneously, the splicing pattern is affected in a predictable way. Moreover, we demonstrate that the SA' and SD' mutations alter the oncogenic specificity of the Akv MLV, displayed by a change in the distribution of the diagnoses of the resulting tumors as well as by an induction of tumors of altered specificity such as histiocytic sarcoma and *de novo *diffuse large B cell lymphoma (DLBCL).

## Results

### Identification of a novel exon residing within the *gag *region of Akv MLV

**Figure 1:**
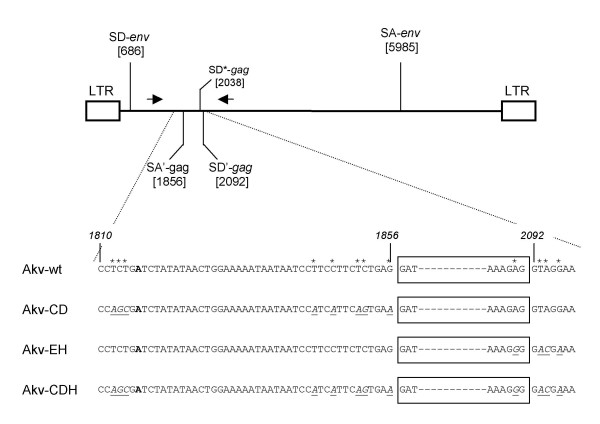
Location of the trapped exon. Upper panel shows the structure of proviral Akv MLV DNA with the positions of the splice sites indicated (SD; splice donor, SA; splice acceptor). Arrows signify the PCR primers used to verify the stability of the introduced mutations. Lower panel shows the positions and types of the introduced mutations, marked by asterisks and underlined. The SA'/SD'-delineated exon is indicated by the box. The boldfaced A in the sequence indicates the presumed branch point.

In order to identify potential alternative splice donor and splice acceptor sites in Akv MLV, exon-trapping was performed using the exon-trapping vector pSPL3 (see Materials and Methods). In short, an exon resulting from usage of the alternative splice acceptor (SA') and either one of two alternative splice donor (SD' or SD*) sites located in the capsid region of *gag *(Fig. 1), was isolated and verified by RT-PCR analyses of RNA isolated from Akv MLV infected cells (data not shown). The size of the exon is 235 bp or 180 bp, depending on the splice donor site used.

### Mutations of the alternative splice sites affect the specificity of the induced tumors

To analyze a possible effect *in vivo *of the novel exon, defined by SA' and SD', three different alternative splice site mutant viruses, Akv-CD, Akv-EH, and Akv-CDH, mutated in either the SA' or SD' site, or in both sites simultaneously, were constructed and injected into newborn mice of the inbred NMRI strain. Fig. 1 shows the precise locations of the synonymous mutations around the trapped exon. Without altering the coding potential of the capsid gene, the mutations affect the branch point site, the pyrimidine region, the conserved splice junction AG and GT dinucleotides, and the fairly well-conserved exonal A at the SD' junction site. The positions of the three intron mutations at the SD' junction site are identical to those in Moloney and Friend MLV described by Audit et al. (1999) [17].

**Figure 2:**
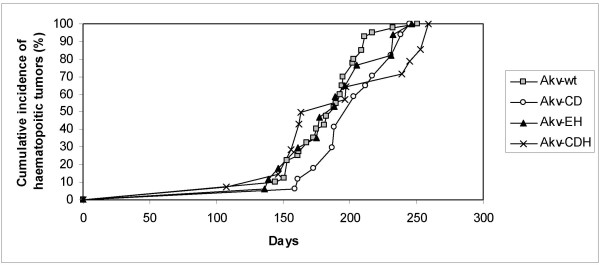
Pathogenicity of Akv and derived splice site mutants in inbred NMRI mice. Shown are the cumulative incidences of tumor development related to age of injected mice (in days).

**Figure 3:**
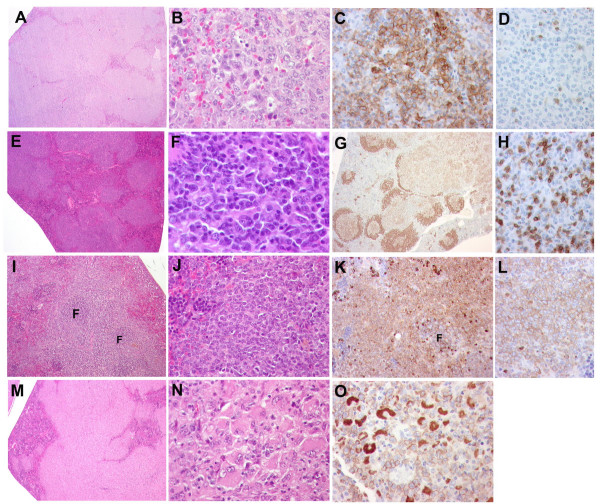
Histopathology of tumors induced by Akv and derived splice site mutants. Representative examples are shown. (A to D) *de novo *diffuse large B-cell lymphoma. (A) Low magnification of a spleen infiltrated by a vaguely nodular lymphoid neoplasia (H&E staining). Magnification, ×25. (B) Higher magnification demonstrates that the neoplasia is composed of a monotonous population of large cells with blastic chromatin, one to three nucleoli and abundant eosinophilic cytoplasm characteristic of centroblasts (H&E staining). Magnification, ×640. (C) Anti-B220 highlights the large neoplastic cells, which are strongly positive (immunohistochemistry). Magnification, ×400. (D) Anti-CD3 shows that only few residual reactive T-cells are present (immunohistochemistry). Magnification, ×400. (E to H) Follicular lymphoma. (E) Low magnification of a spleen infiltrated by a clear nodular lymphoid proliferation (H&E staining). Magnification, ×25 (F) Higher magnification shows a combination of large centroblasts intermingled with small- to medium-sized lymphocytes or centrocytes (H&E staining). Magnification, ×640. (G) Anti-B220 highlights the expansion of the follicles, mainly of the germinal center lymphoid cells (light brown) (immunohistochemistry). Magnification, ×25. (H) Anti-CD3 reveals the presence of abundant reactive T-cells intermingled with the neoplastic B-cells (immunohistochemistry). Magnification, ×400. (I to L) Marginal zone cell lymphoma. (I) Low magnification of a spleen infiltrated by a marginal zone lymphoma. Note that the follicles (F) are small and the cells surrounding these follicles expand and infiltrate the red pulp in a marginal zone pattern (H&E staining). Magnification, ×100. (J) Higher magnification showing that the neoplasia is composed of a monotonous population of small- to medium-sized cells with open fine chromatin, inconspicuous nucleoli and abundant light eosinophilic cytoplasm (H&E staining). Magnification, ×400. (K) Anti-CD79a reveals that the tumor cells in the marginal zone area are strongly positive, whereas the cells in the germinal centers (F) are weakly positive. The opposite staining pattern is seen with anti-B220 (data not shown) (immunohistochemistry). Magnification, ×200. (L) Higher magnification with anti-CD79a shows a uniform membranous positivity of the tumor cells (immunohistochemistry). Magnification, ×400. (M to O) Histiocytic sarcoma. (M) Low magnification of a spleen diffusely infiltrated by a histiocytic sarcoma (H&E staining). Magnification, ×25. (N) Higher magnification shows the presence of large cells with abundant eosinophilic cytoplasm and bland nuclei characteristic of histiocytes (H&E staining). Magnification, ×400. (O) Anti-Mac 3 shows that all tumor cells are positive for this histiocytic marker, both in the cytoplasm and in the cell membrane (immunohistochemistry). Magnification, ×4 Histopathological and immunohistological analyses of tumor tissues.

As can be seen from Fig. 2 and Table 1 the majority of the infected mice (about 90%) developed tumors within 250 days with similar average latency periods of about 200 days for the four types of virus. Histological examination (examples shown in Fig. 3) and diagnosis according to the Bethesda classification [22] revealed that a large proportion (approx. 70%) of the total numbers of tumors could be classified as either follicular B-cell lymphoma (FBL) (13%), diffuse large B-cell lymphoma (DLBCL) progressed from FBL (33%), or plasmacytoma (PCT) (25%) (Table 2). However, the distribution was quite different within the different virus series; thus, almost one quarter of the Akv-wt induced tumors were diagnosed as FBL, while no tumor of the Akv-CD group (p < 0.05) or one tumor each of the Akv-EH or Akv-CDH groups fell into this group. In contrast, within the DLBCL tumors progressed from FBL the frequencies are similar (ranging from 24% to 39%) no matter if the causative virus contained mutated SA' and/or SD' sites or not. In the PCT group it appears that mutating the SA' site significantly impaired the ability of the virus to induce PCT (p < 0.05). On the other hand, this effect was not statistically significant if the SD' site was mutated, and curiously if both sites were mutated, wild-type level for PCT induction was restored.

**Table 1:** Disease latency and frequency

**Virus**	**Average latency period (days)**	**Frequency of mice developing hematopoitic tumors**
**Akv-wt**	184 ± 26	40/40
**Akv-CD**	201 ± 30	17/19
**Akv-EH**	184 ± 34	17/18
**Akv-CDH**	190 ± 46	14/16

**Table 2:** Frequency and latency of induced tumors

**Virus**	**FBL**	**DLBCL (progression from FBL)**	***De novo *DLBCL#**	**PCT**	**SMZL**	**DLBCL (progression from SMZL)**	**SBL**	**PTLL**	**STL**	**Histiocytic sarcoma**
**Akv-wt**	9/40 (23%)	13/40 (33%)	0/40 (0%)	13/40 (33%)	3/40 (8%)	0/40 (0%)	2/40 (5%)	0/40 (0%)	0/40 (0%)	0/40 (0%)
Akv-CD*	0/18 (0%)	7/18 (39%)	0/18 (0%)	1/18 (6%)	5/18 (28%)	1/18 (6%)	0/18 (0%)	0/18 (0%)	0/18 (0%)	4/18 (22%)
**Akv-EH**	1/17 (6%)	4/17 (24%)	6/17 (35%)	3/17 (18%)	0/17 (0%)	1/17 (6%)	0/17 (0%)	0/17 (0%)	1/17 (6%)	0/17 (0%)
**Akv-CDH**	1/14 (7%)	5/14 (36%)	0/14 (0%)	5/14 (36%)	0/14 (0%)	0/14 (0%)	0/14 (0%)	1/14 (7%)	0/14 (0%)	0/14 (0%)

**Total**	11/88 (13%)	29/88 (33%)	6/88 (7%)	22/88 (25%)	8/88 (9%)	2/88 (2%)	2/88 (2%)	1/88 (1%)	1/88 (1%)	4/88 (5%)
**Av. latency period (days)**	188 ± 30	198 ± 31	187 ± 43	180 ± 27	207 ± 20	174 ± 18	153 ± 12	107	146	211 ± 36

Abbreviations: FBL, follicular B cell lymphoma; DLBCL, diffuse large B cell lymphoma; PCT, plasmacytoma; SMZL, splenic marginal zone lymphoma; SBL, small B cell lymphoma; PTLL, precursor T cell lymphoblastic lymphoma; STL, small T-cell lymphoma.

# *De novo *DLBCL refers to Bethesda classification "DLBCL centroblastic"; however, to stress the parallel to human *de novo *lymphomas we use this term.

*In this group one of the 17 mice that developed tumors had two tumors, hence a total number of 18 tumors.

In line with this, the most dramatic effect in general was seen when only the SA' site was mutated as shown for Akv-CD; the tumor incidence of this mutant with respect to splenic marginal zone lymphoma (SMZL) increased from 8% to 28% (p < 0.1) and decreased to 0% as shown for Akv-EH (p < 0.05) and for Akv-CDH (p = 0.5). Moreover, the Akv-CD mutant virus was the only one that displayed a capability for inducing histiocytic sarcoma, a tumor type which has not been observed in any of our previous studies using NMRI mice (inbred or random-bred) infected with Akv, SL3-3, or different derived mutants of these. So in brief, synonymous mutations at the SA' site of Akv MLV markedly altered the oncogenic potential of the virus by significantly impairing the ability to induce both FBL and PCT. Besides, while the development of SMZL was increased by Akv-CD, it was abolished in Akv-EH and Akv-CDH, and most notably, a novel potential for inducing histiocytic sarcoma was established.

The most pronounced effect of mutating the SD' site (Akv-EH) is the frequent occurrence (35%) of diffuse tumors, which according to the Bethesda classification represent DLBCL centroblastic (more than 50% of the infiltrating population is centroblasts). These tumors, where progression is not from either a follicular or a marginal lymphoma, are comparable to the *de novo *lymphomas in humans, and to emphasize this association we have used the term *de novo *DLBCL (Table 2). Strikingly, *de novo *DLBCLs were never observed among the wild-type induced tumors or among the other mutant induced tumors (p < 0.05). The finding of such tumors in mice is rare and could be exploited to understand the molecular changes in *de novo *DLBCL of mice, and eventually a useful mouse model of human *de novo *DLBCL might be generated from this set-up.

Quite unexpectedly, the effect of mutating the SA' and SD' sites simultaneously (Akv-CDH) was the less manifested one. FBL incidence dropped from 23% to 7%; otherwise this mutant in our experimental setting displayed similar tumorigenic potential as the wild-type Akv MLV.

### Conservation of the introduced splice site mutations in the tumors

To determine the stability of the introduced mutations, the regions containing the mutations were PCR amplified from genomic DNA prepared from the induced tumors, using the primers depicted in Fig. 1. The sequences of the amplified fragments confirmed in all cases the integrity of the introduced mutations (data not shown).

### Both single- and double-spliced transcripts are generated *in vivo*

**Figure 4:**
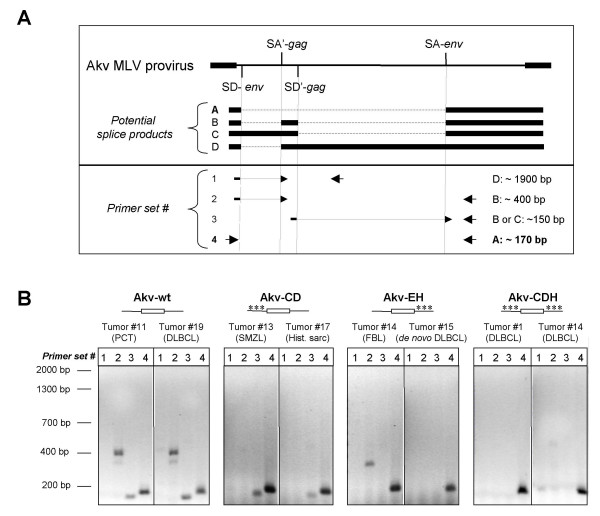
RT-PCR analyses of splice products generated *in vivo*. (A) The structures of the potential splice products A to D are illustrated at the top, with the positions and orientations of the PCR primers (see Materials and Methods) from the four primer sets depicted below. The predicted origins and sizes of the amplified fragments are given at the right. (B) Shown are examples from each series of amplified RT-PCR products visualized on ethidium bromide-stained agarose gels. The employed primer sets (#1 to #4) are listed above the lanes. Size markers are indicated at the left.

The observed effect of the mutated splice sites on the oncogenic properties advocates that RNA splicing by means of the alternative SA' and SD' sites does indeed take place *in vivo*. To clarify and confirm the identity of the produced transcripts, the splice pattern in tumor tissues (and for comparison in NIH 3T3 cells infected with the same four viruses) was analyzed. RNA from the individual end-stage tumors (or from virally infected cells) was isolated, and conventional RT-PCRs were performed with primers designed in such a way that it should be possible to identify all four potential splice products using 4 different primer sets as shown in Fig. 4A.

With a few exceptions, all tumors were analyzed, and sequences of the amplified RT-PCR products determined to validate the specificity of the fragments (data not shown). Representative results from each virus series are shown in Fig. 4B. In all cases, PCR products representing splice product A (the regular *env *transcript; primer set #4) was observed, which implies that damage of the alternative splice sites, SA' and SD', does not impair the production of the regular single-spliced *env *RNA. Concerning splice product D (primer set #1) it was never amplified, neither from tumor tissues nor from cell culture studies, strongly indicating that this is not a *bona fide *transcript. The lack of detection of product D is unlikely to result from a technical PCR-problem since the two primers have been validated in other PCRs.

For the Akv wild-type induced tumors, RT-PCR products representing the double-spliced product B (primer set #2), and fragments of expected size amplified by primer set #3, indicative of splice product B or C, were observed in all cases. As would be expected primer set #2, which is dependent on an intact SA' site, did not result in any amplification products using RNA from Akv-CD or Akv-CDH tumors. Surprisingly however, in five out of 14 analyzed Akv-EH tumor samples (represented by Akv-EH tumor no. 14 in Fig. 4B), a product slightly smaller than that of transcript version B was amplified. The subsequent sequence analyses revealed that the alternative splice donor site SD* (depicted in Fig. 1) in these cases consistently had been used, resulting in the generation of a splice product equivalent in structure to product B, however 54 nucleotides shorter. No correlation between tumor cell specificity and usage of the SD* site could be observed, since the five tumor samples originated from FBL, DLBCL progressed from FBL, *de novo *DLBCL, and the single case of STL (small T-cell lymphoma). The presence of the same splice product from the SD* site was verified by sequence analysis of RT-PCR products derived from tumors induced by the wild-type virus in some cases, although the product was consistently less prominent than product B.

Transcript C corresponds to the single-spliced transcript of 4.4 kb, which previously has been reported to be produced by both Friend and Moloney MLV using the SD' together with the canonical *env *SA' site [18,19]. Our RT-PCR results confirm the existence of this single-spliced transcript, since products of the expected size were always amplified with primer set #3 using RNA from Akv-CD tumors (Fig. 4B), whereas product B (primer set #2) was never amplified in this material.

In summary, by means of the alternative splice sites that define the novel *gag *exon, both a single-spliced transcript C as well as a novel double-spliced transcript B is produced *in vivo*, and when these alternative splice sites are destroyed, the splicing pattern is changed concordantly.

**Figure 5:**
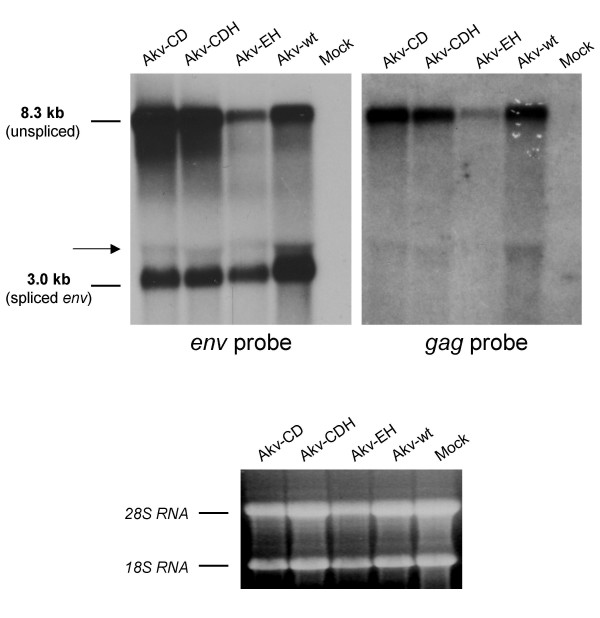
Northern blot hybridizations with an ecotropic specific *env *probe and a *gag *probe of RNA isolated from NIH 3T3 cells chronically infected with the viruses listed above each lane. The sizes of the full-length transcript (unspliced) and the single-spliced *env *transcript are indicated at the left. The arrow indicates splice product C. For verification of integrity and concentration of the loaded RNA, the original ethidium bromide stained agarose gel exposing 18S and 28S rRNAs is shown below.

The same RT-PCR analyses were performed for NIH 3T3 cells infected with the four viruses, which led to the same splice pattern (data not shown). In addition, Northern blot hybridizations with an ecotropic *env*-probe and with a probe covering the novel SA'-SD' defined exon in *gag *were performed with RNA isolated from these cells (Fig. 5). Besides the expected hybridization patterns of prominent bands of full-length (*env *and *gag *probe) and *env *mRNA (only *env *probe) sizes, a weaker band of a size corresponding to splice product C (4.4 kb) was detected with both probes. No distinct band corresponding to spliced RNA B was observed, suggesting a very low level of production and/or significant messenger instability.

### Provirus integration site analyses

In order to identify a possible connection between specific retroviral integration sites (RIS) and specific diagnostic tumor types, provirus integration sites from the majority of the induced tumors were isolated and sequenced. We have then by subsequent homology searches of the mouse genome databases identified 240 unambiguous integration sites (Table 3). These integration site sequences represent tumors from 30 out of 40 (104 sequences), 14 out of 19 (46 sequences), 14 out of 18 (51 sequences), and 11 out of 16 (39 sequences) mice infected by Akv-wt, Akv-CD, Akv-EH, and Akv-CDH, respectively. This corresponds to an average of 3.6 integrations per analyzed tumor. Based on the searches in the UCSC database [23], and the Mouse Retrovirus Tagged Cancer Gene Database, RTCGD [24,25], both version mm8, 111 novel RISs were identified. In an attempt to pick up candidate cancer genes that might be associated with specific tumor diagnoses, we looked for common integration sites (CISs), which would infer such genes [25,26]. Hence, we compared the integration sites with each other as well as with previously defined RISs in RTCGD. In principle, using the recommendations from RTCGD with a window size of 100 kb, 50 kb, and 30 kb for CISs with 4 (or more), 3, or 2 insertions, respectively, we were hereby able to define 35 novel common integration sites (CISs) (Table 3). Just a single one of these could be correlated with a specific diagnosis and with a specific virus, since in two independently Akv-wt induced plasmacytomas a definite region of chromosome 15 was targeted. However, this region does not contain genes/RefSeqs within a 100 kb distance from the integrated proviruses, so for the present we cannot predict what – if any – candidate gene(s) that might have been influenced by the integrated proviruses. For the remaining 34 CISs more than one virus and more than one tumor diagnosis were implicated, which implies that no straightforward association between target gene (and/or causative virus) and tumor type can be deduced.

**Table 3:** Positions of integrated proviruses in tumor DNA

**#**	**Virus**	**Diagnosis**	**Chromosome**	**Position (mm8)**	**Gene/RefSeq^a^**	**No. of hits in RTCGD (mm8)**	**Novel RISs^b^**	**Novel CISs^c^**
1	Akv-EH	DLBCL (from FBL)	1	24641886	Lmbrd1	0	1	-
2	Akv-EH	DLBCL (from FBL)	1	36406157	Cnnm4	0	1	-
3	Akv wt	DLBCL (from FBL)	1	78743292	Kcne4	0	1	-
4	Akv-EH	PCT	1	82855932	Slc19a3	0	1	-
5	Akv wt	FBL	1	93014894	Ramp1	5	-	-
6	Akv-CD	*n.d.*	1	93022552				
7	Akv-EH	Lymphoma, NOS	1	120226476	AK080782	0	1	-
8	Akv-CD	DLBCL (from FBL)	1	130341056	Cxcr4	3	-	-
9	Akv wt	PCT	1	135878316	Fmod/Btg2	8	-	-
10	Akv wt	DLBCL (from FBL)	1	135882183				
11	Akv-CD	Abscess	1	139604557	---	1	-	1
12	Akv wt	PCT	1	144940508	---	0	1	-
13	Akv wt	PCT	1	163725782	AK029097	0	1	-
14	Akv wt	FBL	1	173476364	Slamf7	0	1	-
15	Akv wt	PCT	1	174350588	Tagln2/AK006449	2	-	-
16	Akv wt	DLBCL (from FBL)	1	182219328	MGC68323/AK038867	2	-	-
17	Akv-CD	SMZL	2	11542293	Il2ra	4	-	-
18	Akv-CDH	DLBCL (from FBL)	2	13133178	Rsu1	0	1	-
19	Akv-CDH	PCT	2	35270244	Ggta1	5	-	-
20	Akv wt	DLBCL (from FBL)	2	44741201	Gtdc1	0	1	-
21	Akv wt	DLBCL (from FBL)	2	46263959	---	0	1	-
22	Akv wt	DLBCL (from FBL)	2	71667822	Itga6/Pdk1	0	1	-
23	Akv wt	DLBCL (from FBL)	2	90883313	Slc39a13/Sfpi1	6	-	-
24	Akv wt	SMZL	2	90883476				
25	Akv wt	PCT	2	102668507	Cd44	0	1	-
26	Akv wt	DLBCL (from FBL)	2	102782324	Pdhx	0	1	-
27	Akv wt	FBL	2	118352393	Pak6	0	1	-
28	Akv wt	FBL	2	119028536	Spint1	0	1	-
29	Akv-CDH	Lymphoma, NOS	2	120301032	Zfp106	1	-	1*
30	Akv-CDH	PTLL	2	128875013	Slc20a1	0	1	-
31	Akv-CDH	Lymphoma, NOS	2	129283153	Ptpns1	1	-	1
32	Akv-EH	PCT	2	131711284	Rassf2	0	1	-
33	Akv-CDH	DLBCL (from FBL)	2	158379688	Ppp1r16b	4	-	-
34	Akv wt	SMZL	2	164051325	Slpi	0	1	-
35	Akv wt	DLBCL (from FBL)	2	169860192	Zfp217	5	-	-
36	Akv wt	FBL	3	22265638	Tbl1xr1	0	1	-
37	Akv-CDH	PCT	3	27464311	Aadacl1	0	1	-
38	Akv-CDH	DLBCL (from FBL)	3	30203814	Evi1	5	-	-
39	Akv-CDH	PCT	3	30203870				
40	Akv-CDH	Lymphoma, NOS	3	76043446	Golph4	0	1	-
41	Akv-EH	DLBCL (from FBL)	3	79339620	---	0	1	-
42	Akv wt	FBL	3	90334704	Slc39a1	0	1	-
43	Akv-CD	DLBCL (from FBL)	3	96900321	Cd160	0	1	-
44	Akv wt	SMZL	3	98031475	LOC433632	13	-	-
45	Akv-EH	PCT	3	98041399				
46	Akv-CD	SMZL	3	98043109				
47	Akv-CDH	DLBCL (from FBL)	3	98043150				
48	Akv wt	PCT	3	98043377				
49	Akv wt	DLBCL (from FBL)	3	98043659				
50	Akv-CD	SMZL	3	98064421				
51	Akv-CDH	DLBCL (from FBL)	3	98127957	Notch2	8	-	-
52	Akv-EH	PCT	3	108214198	Ampd2	0	1	-
53	Akv-CD	SMZL	3	115828351	Dph5	1	-	1
54	Akv wt	DLBCL (from FBL)	3	131582947	Papss1	1	-	-
55	Akv wt	SBL	3	145870070	Bcl10	3	-	-
56	Akv wt	DLBCL (from FBL)	3	146091393	Mcoln2	0	1	-
57	Akv-EH	DLBCL (from FBL)	3	157996860	Lrrc40	0	1	-
58	Akv wt	SBL	4	8842182	BC034239	1	-	1
59	Akv-CDH	PCT	4	11915327	AK132816	0	1	-
60	Akv-CD	DLBCL (from FBL)	4	32560128	Bach2	14	-	-
61	Akv wt	FBL	4	32611866				
62	Akv-CD	*n.d.*	4	32619341				
63	Akv wt	PCT	4	32702311				
64	Akv-CD	Histiocytic sarcoma	4	44734542	Pax5	4	-	-
65	Akv wt	SMZL	4	55369934	Rad23b	1	-	1
66	Akv-CDH	Plasma cell prolif.	4	57933461	Akap2	0	1	-
67	Akv-CD	DLBCL (from FBL)	4	97386196	Nfia/D90173	2	-	-
68	Akv-EH	DLBCL (from FBL)	4	132220004	Fgr	3	-	-
69	Akv wt	SMZL	4	134699599	Dscr1l2	0	1	-
70	Akv-CDH	PCT	4	138050107	Pla2g2d	0	1	-
71	Akv wt	FBL	5	39921672	Hs3st1	0	1	-
72	Akv-CDH	Lymphoma, NOS	5	65179064	Tlr1	1	-	1
73	Akv-CD	Histiocytic sarcoma	5	75074642	---	0	1	-
74	Akv-CDH	PTLL	5	107966364	Gfi1	78	-	-
75	Akv-CDH	PCT	5	121838640	Aldh2	0	1	-
76	Akv-CDH	DLBCL (from FBL)	5	141077075	Gna12	4	-	-
77	Akv wt	DLBCL (from FBL)	6	29717975	4631427C17Rik	0	1	-
78	Akv-EH	*de novo *DLBCL	6	40821642	BC048599	0	1	-
79	Akv-CDH	Lymphoma, NOS	6	40955151	2210010C04Rik	0	1	-
80	Akv-CDH	PCT	6	54425558	Scrn1	0	1	-
81	Akv wt	FBL	6	72441620	BC100525	2	-	-
82	Akv wt	PCT	6	84016089	Dysf	0	1	-
83	Akv-CDH	Lymphoma, NOS	6	88923549	Gpr175	0	1	-
84	Akv-EH	DLBCL (from SMZL)	6	99153396	Foxp1	1	-	1*
85	Akv-CDH	Lymphoma, NOS	6	113010477	Thumpd3	1	-	1
86	Akv-CD	DLBCL (from FBL)	6	120535110	Cecr5	3	-	-
87	Akv-CD	Histiocytic sarcoma	6	136905161	Arhgdib	0	1	-
88	Akv-CD	PCT	6	145079282	Lrmp	4	-	-
89	Akv-CDH	Plasma cell prolif.	7	18841894	Apoc4	0	1	-
90	Akv wt	FBL	7	24263292	Xrcc1	0	1	-
91	Akv wt	PCT	7	28498093	5830482F20Rik	0	1	-
92	Akv-EH	*de novo *DLBCL	7	28691963	Map4k1	1	-	1
93	Akv-EH	PCT	7	29760852	Zfp14	0	1	-
94	Akv-CD	Histiocytic sarcoma	7	30746023	Fxyd5	0	1	-
95	Akv wt	PCT	7	45003648	Flt3l	0	1	-
96	Akv wt	DLBCL (from FBL)	7	66812108	Adamts17	0	1	-
97	Akv wt	DLBCL (from FBL)	7	73481277	---	7	-	-
98	Akv-CD	SMZL	7	76335733	D430020F16	0	1	-
99	Akv-EH	DLBCL (from FBL)	7	79083435	Mfge8	0	1	-
100	Akv-EH	PCT	7	80764333	AK034740	0	1	-
101	Akv wt	PCT	7	82574727	Eftud1	1	-	1*
102	Akv-EH	*de novo *DLBCL	7	99422958	Arrb1/mmu-mir-326	0	1	-
103	Akv wt	SBL	7	113972740	Rras2/Copb1	32	-	-
104	Akv-CDH	DLBCL (from FBL)	7	121248057	AK043969	0	1	-
105	Akv-EH	*de novo *DLBCL	7	126957227	Cd2bp2	2	-	-
106	Akv wt	DLBCL (from FBL)	7	132147451	4631426J05Rik	0	1	-
107	Akv-CD	DLBCL (from FBL)	7	144741729	Ccnd1	21	-	-
108	Akv-CD	SMZL	8	8401964	---	0	1	-
109	Akv-EH	DLBCL (from FBL)	8	10980425	---	4	-	-
110	Akv wt	FBL	8	35575516	Dctn6	0	1	-
111	Akv wt	PCT	8	37098099	---	0	1	-
112	Akv-CD	SMZL	8	74527100	Pgls	3	-	-
113	Akv wt	DLBCL (from FBL)	8	98522228	Gins3	0	1	-
114	Akv wt	SBL	8	112753504	BC027816	0	1	-
115	Akv-EH	*de novo *DLBCL	8	118119184	Wwox	1	-	1
116	Akv-EH	Lymphoma, NOS	8	123611854	Irf8	1	-	-
117	Akv-EH	DLBCL (from FBL)	8	126312417	Tubb3/Mela	1	-	1
118	Akv wt	DLBCL (from FBL)	8	126312418				
119	Akv wt	PCT	9	46235263	---	0	1	-
120	Akv-CD	Abscess	9	86514607	A330041J22Rik	0	1	-
121	Akv-CDH	PCT	9	104103363	Acpp	1	-	1
122	Akv-EH	FBL	9	115327386	---	0	1	-
123	Akv wt	PCT	9	117143474	Rbms3	0	1	-
124	Akv-EH	PCT	10	5056902	Syne1	1	-	-
125	Akv wt	SBL	10	7626524	Map3k7ip2	1	-	1
126	Akv wt	FBL	10	19681745	Map3k5	2	-	-
127	Akv wt	PCT	10	43100419	Pdss2	0	1	-
128	Akv-EH	PCT	10	59295561	Dnajb12	2	-	-
129	Akv-CD	Histiocytic sarcoma	10	75678805	Prmt2	0	1	-
130	Akv wt	FBL	10	77412375	Pfkl	0	1	-
131	Akv wt	DLBCL (from FBL)	10	8009951	BC058238	2	-	-
132	Akv wt	DLBCL (from FBL)	10	84366577	Ric8b	0	1	-
133	Akv-EH	*de novo *DLBCL	10	87574719	BC070476	0	1	-
134	Akv-EH	*de novo *DLBCL	10	92501677	Pctk2	1	-	-
135	Akv-CD	*n.d.*	10	123752010	---	0	1	-
136	Akv-CDH	PCT	11	3236433	1500004A08Rik	0	1	-
137	Akv wt	PCT	11	5331124	AK133342	0	1	-
138	Akv wt	DLBCL (from FBL)	11	23587218	4933435A13Rik	4	-	-
139	Akv-CD	DLBCL (from FBL)	11	23587651				
140	Akv wt	PCT	11	32443618	Stk10	1	-	1
141	Akv wt	PCT	11	46693128	Timd4	0	1	-
142	Akv-CD	PCT	11	51687729	Phf15	0	1	-
143	Akv-EH	*de novo *DLBCL	11	62648918	Trim16	0	1	-
144	Akv wt	PCT	11	67380781	Gas7	0	1	-
145	Akv-EH	*de novo *DLBCL	11	74962635	Smg6	9	-	-
146	Akv-EH	*de novo *DLBCL	11	78821537	Ksr1	0	1	-
147	Akv-EH	PCT	11	86862765	Gdpd1	0	1	-
148	Akv wt	DLBCL (from FBL)	11	95031687	Tac4	0	1	-
149	Akv-EH	*de novo *DLBCL	11	102249514	Grn	0	1	-
150	Akv wt	DLBCL (from FBL)	11	102990732	Fmnl1	2	-	-
151	Akv wt	DLBCL (from FBL)	11	106946907	Nol11	0	1	-
152	Akv-CD	Abscess	11	107232889	Pitpnc1	1	-	1
153	Akv-EH	PCT	11	116126666	Exoc7	0	1	-
154	Akv-CDH	Lymphoma, NOS	11	118058083	Pscd1	1	-	1*
155	Akv-CD	SMZL	12	3288080	Rab10	0	1	-
156	Akv wt	PCT	12	13172238	Ddx1	0	1	-
157	Akv-CD	Abscess	12	56600484	Garnl1	0	1	-
158	Akv-EH	*de novo *DLBCL	12	77286036	Zbtb25	1	-	1
159	Akv wt	DLBCL (from FBL)	12	80214408	AK132344	1	-	1
160	Akv wt	DLBCL (from FBL)	12	86569587	Batf	2	-	-
161	Akv-CD	DLBCL (from FBL)	12	113688885	BC004786	5	-	-
162	Akv wt	PCT	13	24453563	Cmah	1	-	1*
163	Akv-CDH	DLBCL (from FBL)	13	28624333	---	0	1	-
164	Akv wt	PCT	13	28727388	---	3	-	-
165	Akv-CDH	DLBCL (from FBL)	13	28764182				
166	Akv-CDH	DLBCL (from FBL)	13	28950798	Sox4	79	-	-
167	Akv-CD	DLBCL (from FBL)	13	28950905				
168	Akv-EH	*de novo *DLBCL	13	28955972				
169	Akv wt	DLBCL (from FBL)	13	28958981				
170	Akv-EH	DLBCL (from FBL)	13	30695323	Dusp22	4	-	-
171	Akv-CD	Histiocytic sarcoma	13	30727958				
172	Akv wt	PCT	13	31914670	Gmds	1	-	1
173	Akv-CD	SMZL	13	36214883	Fars2	0	1	-
174	Akv wt	PCT	13	37804150	Rreb1	8	-	-
175	Akv-CD	Histiocytic sarcoma	13	38701503	Eef1e1	1	-	-
176	Akv-CD	DLBCL (from FBL)	13	43205444	Gfod1	1	-	1*
177	Akv-CD	SMZL	13	63488458	Fancc	4	-	-
178	Akv wt	DLBCL (from FBL)	13	84050271	Mef2c	11	-	-
179	Akv-CDH	DLBCL (from FBL)	14	6779744	Dnase1l3	1	-	1
180	Akv-CDH	Lymphoma, NOS	14	24439077	Rai17	10	-	-
181	Akv-CDH	PCT	14	25348097	Slmap	1	-	1
182	Akv-EH	Lymphoma, NOS	14	29013598	Cacna1d	0	1	-
183	Akv wt	SBL	14	30491074	Btd	0	1	-
184	Akv wt	DLBCL (from FBL)	14	59481891	---	0	1	-
185	Akv wt	FBL	14	59637396	AK151394	3	-	-
186	Akv-EH	*de novo *DLBCL	14	63115780	Msra	1	-	1*
187	Akv wt	PCT	14	72420139	Sucla2	0	1	-
188	Akv wt	SMZL	14	113921088	microRNA cluster	2	-	-
189	Akv wt	FBL	15	57752356	BC066830	0	1	-
190	Akv-CD	SMZL	15	58238320	15Ertd621e	0	1	-
191	Akv wt	PCT	15	61240727	---	0	1	1
192	Akv wt	PCT	15	61240729				
193	Akv wt	FBL	15	73548838	Gpr20/Ptp4a3	6	-	-
194	Akv-EH	DLBCL (from FBL)	15	79567673	Unc84b	4	-	-
195	Akv wt	DLBCL (from FBL)	15	79728346	Apobec3	0	1	-
196	Akv-EH	Plasma cell prolif.	15	90445122	Cpne8	1	-	-
197	Akv wt	FBL	16	23906462	Bcl6	5	-	-
198	Akv-EH	*de novo *DLBCL	16	24049008	---	5	-	-
199	Akv-CD	DLBCL (from FBL)	16	24086635				
200	Akv-EH	DLBCL (from FBL)	16	24101470				
201	Akv-EH	Lymphoma, NOS	16	24152772				
202	Akv wt	FBL	16	24178252				
203	Akv wt	DLBCL (from FBL)	16	32049420	Lrrc33	3	-	-
204	Akv-CD	*n.d.*	16	52388367	Alcam	1	-	1
205	Akv wt	PCT	17	6624877	Vil2	3	-	-
206	Akv wt	PCT	17	11630998	Park2	1	-	1*
207	Akv wt	DLBCL (from FBL)	17	36597927	2410137M14Rik	0	1	-
208	Akv wt	FBL	17	49537999	2310015N21Rik	1	-	1*
209	Akv-EH	DLBCL (from FBL)	17	63705275	Fert2	0	1	-
210	Akv-CD	SMZL	17	71389894	Kntc2	0	1	-
211	Akv-CDH	PTLL	17	74614066	Birc6	1	-	-
212	Akv wt	PCT	18	11242422	---	0	1	-
213	Akv-CD	SMZL	18	12368360	Npc1	1	-	1
214	Akv-CD	SMZL	18	36016553	Cxxc5	3	-	-
215	Akv-CDH	DLBCL (from FBL)	18	39790170	---	0	1	-
216	Akv wt	FBL	18	42916219	Ppp2r2b	0	1	-
217	Akv-EH	*de novo *DLBCL	18	60930595	Ii/Cd74	0	1	1
218	Akv-EH	DLBCL (from FBL)	18	60930793				
219	Akv-CD	DLBCL (from FBL)	18	60930833				
220	Akv wt	PCT	18	60931233				
221	Akv-CDH	PCT	18	60934151				
222	Akv wt	DLBCL (from FBL)	18	61103722	Camk2a	0	1	-
223	Akv-EH	PCT	18	65601468	Malt1	1	-	-
224	Akv wt	DLBCL (from FBL)	18	67824697	Ptpn2	0	1	-
225	Akv wt	FBL	19	11607493	Ms4a4d	0	1	-
226	Akv wt	DLBCL (from FBL)	19	34362400	Fas	2	-	-
227	Akv-EH	*de novo *DLBCL	19	37505125	Hhex/Exoc6	44	-	-
228	Akv-CD	DLBCL (from FBL)	19	37532944				
229	Akv-EH	*de novo *DLBCL	19	37537038				
230	Akv-CD	SMZL	19	37560104				
231	Akv-CD	Histiocytic sarcoma	19	37560104				
232	Akv-EH	PCT	19	43474871	Cnnm1	0	1	-
233	Akv-CDH	PCT	19	47583866	Obfc1	0	1	-
234	Akv wt	DLBCL (from FBL)	19	47963460	AK014581	0	1	-
235	Akv wt	PCT	19	53994934	Shoc2	0	1	-
236	Akv wt	FBL	19	55633727	Vti1a	1	-	1*
237	Akv wt	FBL	X	103293531	P2ry10	0	1	-
238	Akv-CDH	Lymphoma, NOS	X	109985132	Dach2	1	-	1
239	Akv-EH	DLBCL (from FBL)	X	129928954	Btk	0	1	-
240	Akv wt	DLBCL (from FBL)	X	162452646	Tmsb4x	1	-	1

^a^The gene (or RefSeq) closest to the integrated provirus is given (UCSC, mouse mm8 assembly). --- indicates that the distance to the closest gene/RefSeq is more than 100 kb.

^b^For each insertion, it is indicated, based on RTCGD (mm8), whether a novel RIS has been defined.

^c^For each insertion, it is indicated, based on RTCGD (mm8), whether a novel CIS has been defined. The definition follows the recommendations from RTCGD with a window size of 100 kb, 50 kb, and 30 kb for CISs with 4 (or more), 3, or 2 insertions, respectively. * indicates an exception from this rule, if two integration sites are found within the same gene/RefSeq.

*n.d.*, not determined.

In six cases, the same chromosomal locus was targeted several times. These cases include *Bach2 *(hit 4 times), *Sox4 *(hit 4 times), *Hhex *(hit 5 times), *Ii *(hit 5 times), a region of chr. 16 not containing any genes/RefSeqs within a distance of 100 kb from the integration sites (hit 5 times), and *LOC433632 *(hit 7 times). Five of these integration sites were already registered in RTCGD, only the *Ii *locus define a completely novel RIS/CIS. This latter finding may suggest that *Ii *targeting is strongly associated with the applied model system (virus/mouse strain)[27]. We also note that an integration has taken place within the first intron of *Stk10 *in a plasmacytoma induced by Akv-wt (Table 3, #140), which appears to be in conflict with the work of Shin *et al.*, 2004 [28] where *Stk10 *was described as a SMZL specific candidate gene.

Finally, we examined if specific regions of the targeted gene/RefSeq have been favored with respect to orientation and position of the integrated provirus. We have recently reported of differences between Akv MLV and an enhancer mutant hereof, Akv1-99, in their patterns of proviral insertions around host transcription units in the induced tumors [29]. In line with this, it might be envisioned that destroying the alternative splice sites of the virus could lead to a different pattern of integration site selection during tumorigenesis; *e.g*. it might be speculated if particular positions relative to the target gene somehow would facilitate gene deregulation dependent on the presence or absence of intact SA' and/or SD' sites. Accordingly, we allocated each individual integration site position and orientation to a defined region, *i.e. *either upstream, within the promoter, within 1. intron, within last intron, within all other introns, within exons, or downstream of the target gene (Table 4). Eleven cases in total were excluded as they were positioned – almost with the same distance – in between two target genes, upstream of one and downstream of the other. As seen in table 4, no clear differences with respect to the four viruses were observed, signifying that mutations of the alternative splice sites do not have major effect on the ability of the Akv MLV to affect the target gene/RefSeq from certain positions and/or orientations. It might be worth to notice that the overall picture shows that about half of all integrations are found within introns, and among these there appear to be a tendency for a provirus orientation opposite to that of the target gene. In these cases the formation of chimeric RNA species by promoter insertion and/or splicing would not be predicted.

**Table 4:** Frequency of proviral insertions within defined genomic regions

**Virus**	**Upstream^a^**		**Promoter^b^**		**1. intron**		**Internal intron**		**Last intron**		**Downstream^c^**		**Exon**		**Outside^d^**
		
	**+**	**-**	**+**	**-**	**+**	**-**	**+**	**-**	**+**	**-**	**+**	**-**	**+**	**-**	
**Akv-wt**	2/104 2%	7/104 7%	5/104 5%	3/104 3%	9/104 9%	16/104 15%	6/104 6%	17/104 16%	2/104 2%	1/104 1%	5/104 5%	8/104 8%	2/104 2%	1/104 1%	11/104 10%
**Akv-CD**	3/46 7%	6/46 13%	1/46 2%	0/46 0%	3/46 7%	5/46 11%	2/46 4%	8/46 17%	0/46 0%	0/46 0%	4/46 9%	6/46 13%	1/46 2%	0/46 0%	5/46 11%
**Akv-EH**	3/51 6%	4/51 8%	1/51 2%	0/51 0%	4/51 8%	6/51 12%	2/51 4%	11/51 22%	2/51 4%	0/51 0%	7/51 14%	1/51 2%	2/51 4%	2/51 4%	6/51 12%
**Akv-CDH**	1/39 3%	2/39 5%	2/39 5%	3/39 8%	7/39 18%	3/39 8%	3/39 8%	5/39 13%	0/39 0%	1/39 3%	5/39 13%	4/39 10%	0/39 0%	0/39 0%	3/39 8%

**Total**	9/240 4%	19/240 8%	9/240 4%	6/240 3%	23/240 10%	30/240 13%	13/240 5%	41/240 17%	4/240 2%	2/240 1%	21/240 9%	19/240 8%	5/240 2%	3/240 1%	25/240 10%

^a ^Within 3–100 kb upstream of target gene/RefSeq

^b ^Within 0–3 kb upstream of exon1 of target gene/RefSeq

^c ^Within 100 kb downstream of target gene/RefSeq

^d ^Proviral integrations > 100 kb away from gene/RefSeq

+ or - denotes the orientation of the integrated provirus relative to the target gene/RefSeq.

## Discussion

**Figure 6:**
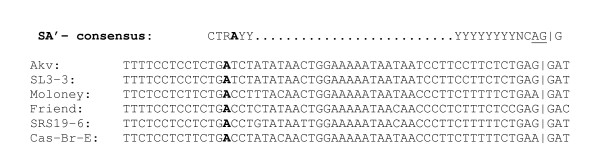
DNA sequence alignment around the Akv MLV SA' site in the capsid-coding region of a series of different ecotropic MLVs. The 3' splice acceptor site consensus sequences are shown on top, with the border of the novel *gag *exon indicated by a vertical line. The boldfaced A in the sequence indicates the presumed branch point.

We have in the B-lymphomagenic Akv MLV identified a novel exon, which is defined by the alternative splice acceptor (SA') and the splice donor (SD') sites located in the capsid encoding region. While previous studies of Moloney and Friend MLV have demonstrated production of a 4.4 kb transcript using the same SD' site together with the canonical *env *SA site, this is the first report demonstrating the existence of an alternative SA' site and production of a double-spliced transcript during the life cycle of a replication-competent simple retrovirus. Yet it remains to be investigated how widespread this competence is. An alignment between six murine retroviruses shows that the conserved splice junction dinucleotide AG is present neither in Cas-Br-E nor in Moloney MLV, although the region in general is well-conserved (Fig. 6).

We did not perform detailed analyses of the influence of the splice site mutations on the viral replication. However, since the same number (10^5 ^to 10^6^) of infectious virus particles, as measured by infectious center assays, were injected from each virus series, and since the mutant viruses induced tumors with comparable incidences and latencies as the wild-type virus, it is not likely that the mutations had imposed severe weakening on the *in vivo *spreading capability. Hence the observed shifts in specificity of the induced tumors most likely are a direct result of the introduced mutations. However, we note that Houzet et al. [19] observed a reduction in titer of SD'-mutants of Friend virus.

The *in vivo *significance of the alternative splice sites was exposed by a change of the oncogenic properties of Akv MLV, when synonymous mutations destroying the SA' site, the SD' site, or both sites simultaneously were introduced. First and foremost, the obvious capability of Akv MLV to induce follicular B cell lymphoma was seriously weakened, when one or both of the alternative splice sites were mutated, suggesting that this competence relies on intact SA' and SD' sites and a proper balance between all the produced transcripts (one full-length, two single-spliced, and one double-spliced). The integrity of Akv MLV seems fundamental for its capability to induce FBL; thus we just reported that Akv MLVs with mutated enhancer sequences retained the ability to induce tumors of B-cell type, but altered specificities were observed, including an impaired ability of FBL induction [11]

A more complex picture was observed regarding the strong predisposition of Akv MLV for plasmacytoma induction. This predisposition was affected significantly only if the SA' alone was mutated. Thus, if the SD' site was mutated along with SA', wild-type potential was restored. This may indicate that the ability to induce plasmacytoma is dependent on a fine-tuned balance between the alternative single-spliced and double-spliced transcripts. If no double-spliced transcript is produced, while the single-spliced 4.4 kb transcript still is, as is the case for the SA' mutant, the single-spliced transcript somehow seems to be related with a barrier for plasmacytoma induction. On the other hand, if both transcripts are produced (Akv-wt) or none of them are produced (the SA'/SD' double mutant) the virus will hold a potential for inducing plasmacytomas. This is in line with the overall observation that the most pronounced effects were observed when the SA' or SD' splice sites were mutated individually, while the outcome of infection with the SA'/SD' double mutant in essence, except for the capability of FBL induction, was comparable to that of the wild-type virus. It may thus be speculated if a delicate balance between the alternatively single-spliced and double-spliced transcripts is a key determinant for the shift in oncogenic specificity, as demonstrated by the SA' and SD' splice site Akv mutants.

The most striking shifts in specificity observed were the increased tendency to stimulate development of splenic marginal zone lymphoma and the exposure of a novel ability for inducing histiocytic sarcoma for the SA' site mutant. Although we have shown that mutation of the SA' site results in inhibition of generation of the double-spliced product, we are at this point not able to explain or point to any detailed mechanisms underlying the observed changes in specificity. The other remarkable shift in specificity was detected with the SD' mutant, which was the only virus capable of inducing centroblastic DLBCL, *i.e*. tumors for which an origin from the follicle or marginal zone could not be inferred and comparable to *de novo *DLBCL in humans. Moreover, since the exposed potential appeared quite strong (35% of the SD'-mutant induced tumors fell within this diagnosis), and since such tumors are in general rare in mice, this mutant virus may be a helpful starting point tool to create a solid mouse model of human *de novo *DLBCL.

Obviously, the proposed significance of proper balances between the four different transcripts for the observed shifts in tumor specificity may reflect a need for a well-regulated balance between resulting translational products. We did not investigate if novel proteins were produced, but the open reading frames (ORFs) of both the alternatively singly and doubly spliced transcripts clearly reveal a potential for additional proteins to be produced. The gene products of the single-spliced 4.4 kb transcript most probably correspond to the p50 and p60 proteins made from the equivalent Friend MLV transcript [19]. These proteins were produced with translation initiations at two initiation codons (AUG^*gag *^and CUG^*glyco*-*gag*^) in the same ORF and were shown to harbor the N-terminal Gag domain including matrix, p12, and the first 110 amino acids of the capsid in frame with the last 116 amino acids of integrase [19]. Also the smaller double-spliced transcript harbors smaller ORFs providing a scene for even more MLV proteins.

Intragenic elements such as *gag *enhancers have been known for many years in avian retroviruses [30,31]. However, it seems unlikely that a similar element is involved here, since the mutant virus with SA' and SD' sites mutated together was clearly the less affected one. This observation, in concert with the observed consequence on generation of different splice products, more likely suggests that the effect on disease specificity is related to an RNA processing phenomenon rather than an intragenic *gag *determinant with an effect on transcription.

Lymphoma-induction by non-acute murine retroviruses is associated with multiple proviral insertions that affect critical host genes. To achieve such multiple insertions the superinfection resistance caused by Env-expression must be by-passed. One possibility could be that reduced Env-expression caused by mutation of the *gag *splice sites as reported here might favor superinfection and thereby multiple proviral insertions. While we cannot exclude this possibility, our finding of the same number of sequence tags for proviral insertions for the wild-type and mutated viruses gives no immediately support to such a mechanism.

Retroviral insertional mutagenesis has been established as a solid strategy for the identification of candidate cancer-causing genes [6,26,32-34]. Accordingly, in an effort to relate specific genes or pathways with specific diagnoses, splice pattern, or causative virus, we identified a pool of 240 integration sites from which 111 novel RISs and 35 novel CISs were defined. Our analyses did not immediately point to any clear correlations; nevertheless the collection of candidate genes may prove to be a central input in future attempts to understand the exact roles of the different splice transcripts and/or their resulting translational products in hematopoietic differentiation and tumorigenesis.

## Conclusion

We have in the B-lymphomagenic Akv MLV in the *gag *region identified a novel exon, which represents the first example of a doubly spliced gammaretroviral transcript. Mutations of the alternative splice sites that define this novel transcript change the distribution of the different induced tumor phenotypes as well as generate tumors of additional specificities such as *de novo *diffuse large B cell lymphoma and histiocytic sarcoma. Provirus integration site analyses revealing 111 novel RISs and 35 novel CISs did not clearly point to specific target genes or pathways to be associated with specific tumor diagnoses or individual viral mutants. However, the list of potential target genes will be useful for future studies of hematopoietic differentiation and tumorigenesis.

## Methods

### Exon trapping

Exon trapping was performed by using an Exon Trapping System kit (GibcoBRL) in essence according to supplied protocol. In brief, Akv DNA was digested with *Bam*HI or *Bgl*II and all restriction fragments were subcloned into the pSPL3 plasmid, which in addition to sequences necessary for replication and growth in *Escherichia coli *contains SV40 sequences that provide for replication and transcription in COS-7 cells, splicing signals, and a multiple cloning site. Following transformation into *E. coli*, plasmid DNA was isolated and transfected into COS-7 cells. Total RNA was isolated from cultured cells and used for first-strand cDNA synthesis. The cDNA was PCR amplified in two rounds with primers located in the vector exons. The outcome of the PCR amplifications was several different fragments, which were all sequenced. Two of trapped sequences could be verified as exons by RT-PCR analyses of RNA isolated from Akv MLV infected cells. The two trapped exons were defined by the same splice acceptor site (SA', located in the *gag *region, Fig. 1), but by different splice donor sites (SD' and SD*, Fig. 1), and the sizes were 235 bp and 180 bp, respectively.

### Generation of viruses

The mutations of Akv MLV at splice acceptor (SA') and/or splice donor site (SD') sites were introduced by PCR-based oligonucleotide directed mutagenesis using the following primers harboring the wanted mutations (underlined): Mut-C: 5'-CTATATAACTGGAAAAATAATAATCCATCATTCAGTGAAGATCCAGGTAAACT-3', Mut-D: 5'-GGATTATTATTTTTCCAGTTATATAGATCGCTGGAGGAAAACG-3', and Mut-H: 5'-TTGGGATTACACCACCCAAAGGGGACGAAACCACCT-3'. A 720 bp *Bsu*36I – *Bsu*36I fragment harboring the mutations was cloned into the full length parental provirus. The correct sequence of the introduced *Bsu *36I fragment was verified by sequence analysis.

### Pathogenicity experiments

Akv wild-type virus λ623 and the three different alternative splice site mutant viruses, Akv-CD, Akv-EH, and Akv-CDH, mutated in either the SA' or SD' site, or in both sites simultaneously, were injected into newborn mice of the inbred NMRI strain, as described in details [35]. Control mice of the same colony were mock injected with 0.1 mL complete medium. The animals were monitored 5 days per week. Mice were sacrificed and autopsied when showing signs of illness or tumor development. Tumor development was diagnosed on the basis of grossly enlarged lymphoid organs after having reached the size described earlier, which is compatible with lymphoma [36]. Lymphoid tumor tissues and the liver were dissected, stored frozen (-80°C) and/or fixed in formalin for further analysis. Statistical analysis was carried out using the two-tailed Fisher's exact test.

### Histopathological examination and immunohistochemical analysis

Formalin-fixed, paraffin-embedded sections from lymph nodes, thymus, spleen and liver were analyzed. Three-to-five micrometer-thick sections were cut and stained with hematoxylin and eosin (H&E), and when indicated with Giemsa, PAS or chloroacetate esterase. Tumors were classified according to the Bethesda proposals for classification of murine hematopoietic neoplasms [22,37]. Immunohistochemistry was performed on an automated immunostainer (Ventana Medical System, Inc.; AZ, USA), according to the protocol provided by the company with minor modifications. After deparaffinization and rehydration, the slides were placed in a microwave pressure cooker in 0.01 M citrate buffer (pH 6.0), containing 0.1% Tween-20 and heated in a microwave oven at maximum power for 30 min. After cooling in Tris-buffered saline, the sections were incubated with 3% goat or rabbit serum for 20 min. The antibody panel used included CD3, CD79acy, TdT, myeloperoxidase (Dako, Hamburg, Germany), B220/CD45R and MAC3 (BD Pharmingen, NJ, USA). Appropriate positive controls were used to confirm the adequacy of the staining.

### Northern blot analysis

Total cellular RNA was extracted from chronically infected NIH 3T3 cells by Trizol Reagent (Invitrogen), following the manufacturer's recommendations. Approximately 25 μg of RNA from each series (Akv-wt, Akv-CD, Akv-EH, Akv-CDH, and mock-infected cells) was size-fractionated on a 1.2% formaldehyde/agarose gel, and transferred to a nylon filter membrane (Zeta-Probe GT; Bio-Rad) under alkaline conditions (50 mM NaOH). Prehybridzation, hybridization, and washing procedures were according to standard protocol described in the instruction manual from Zeta-Probe GT [(pre)hybridization buffer: 0.25 M sodium phosphate, pH 7.2, 7% SDS, and washing buffers: 20 mM sodium phosphate, ph 7.2, 5% (1%) SDS). The hybridization probes were a ^32^P random priming labeled envelope specific probe (a 330 bp *Sma*I fragment of Akv MLV (positions 6240to 6570) [38]]) and a ^32^P random priming labeled *gag *specific probe covering the novel exon. The *gag *probe was a 380 bp PCR-fragment amplified by the following primers: *gag*-forward: 5'-ATGGTCAGTTGCAGTACTGGCCGT-3' and *gag*-reverse: 5'-TGGGGCTTCGGCCCGCGTTTTGGA-3'. The integrity and concentration of the RNA were confirmed by visual inspection of ethidium bromide-stained 18S and 28S rRNAs.

### PCR and RT-PCR analyses

Genomic DNA was purified from frozen tumor tissues by DNeasy Tissue Kit (Qiagen). Conservation of the introduced mutations was examined by PCR amplifying the region enclosing the mutations and by using the primers depicted in Fig. 1 (primer sequences and positions in Akv provirus: Forward primer, 5'-CCTATGAACCCCCTCCGTGGGTCA-3', nucleotides 1387–1410, and Reverse primer, 5'-TATTAAAGATCCTTTCGGCTTC-3', nucleotides 2412–2390). The resulting PCR products were analyzed by agarose gel electrophoresis, purified, and sequenced with nested sequencing primers (numberings refer to positions in Akv provirus). Forward primer, 5'-CGGGGAGGAGAAGCAGCGGGTGCT-3', nucleotides 1952–1976, and Reverse primer, 5'-GTCCCTAATAATTGCTGGCAAT-3', nucleotides 1942-1921.

For the RT-PCR analyses, total cellular RNA was extracted from tumor tissues or chronically infected NIH 3T3 cells by Trizol Reagent (Invitrogen), following the manufacturer's recommendations. 1–5 μg total RNA was used to make first-strand cDNA by First-Strand cDNA Synthesis Kit (Amersham Biosciences) with an oligo-dT primer. This was followed by standard PCR amplification using four different primer sets, #1 to #4. Primer set #1: Forward primer, 5'-CCGACCCACCGTCGGGAGGAT-3', and reverse primer, 5'-CCTCATCAAACAGGGTGGGACT-3'. Primer set #2: Forward primer, 5'-CCGACCCACCGTCGGGAGGAT-3', and reverse primer, 5'-CACCCACACGGAGTCTCCAAT-3'. Primer set #3: Forward primer, 5'-GATTACACCACCCAAAGAGCTC-3', and reverse primer, 5'-CACCCACACGGAGTCTCCAAT-3'. Primer set #4 (*env *transcript): Forward primer, 5'-TTGGAGACCCCCGCCCAGGGACCACC-3', and reverse primer, 5'-CACCCACACGGAGTCTCCAAT-3'. The resulting RT-PCR products were analyzed by agarose gel electrophoresis, and in most cases purified and sequenced.

### Provirus tagging and analyses

Genomic DNA isolated from the induced tumors was analyzed for provirus integration sites by a splinkerette-based PCR method [26], described in details in [39]. The resulting host/virus junction fragments were sequenced, and the cellular flanking sequences were compared (BLAT search) to the UCSC Genome Browser, version mm8, to determine the chromosomal position of the integrated provirus. To identify possible novel retrovirus integration sites (RISs) and common integration sites (CISs), the individual integration sites were concomitantly matched up to the Retroviral Tagged Cancer Gene Database (RTCGD), version mm8 [24,25]. The definition of a CIS follows the recommendations from RTCGD with a window size of 100 kb, 50 kb, and 30 kb for CISs with 4 (or more), 3, or 2 insertions, respectively. Exception from the recommended window sizes was allowed in a few cases when two (or more) integrations were found within the same gene/RefSeq (Table 3).

### DNA sequencing analysis

Amplified PCR products or purified plasmid preparations were sequenced with the DYEnamic ET terminator cycle sequencing kit (Amersham Pharmacia Biotech), following the manufacturer's recommendations, and reaction products were analyzed on an automated DNA sequencer (Applied Biosystems Inc.).

## Authors' contributions

ABS and AHL carried out the molecular genetic studies (virus generation, exon trapping, PCR and RT-PCR analyses, northern analysis), and ABS drafted the manuscript. SK, LQM, and JS carried out pathogenicity experiments, and histopathological and immunohistochemical analyses. BW and MW carried out provirus tagging analyses. ABS, AHL, and FSP conceived of the study, and participated in its design and coordination. All authors read and approved the final manuscript.
